# Preparation of Suaeda Tea Through Semi-Solid Fermentation Utilizing *Kluyveromyces marxianus*, *Komagataeibacter europaeus*, and *Acetobacter schutzenbachii*: Physicochemical Characteristics, Process Optimization, and Antioxidant Activity

**DOI:** 10.3390/biotech14040083

**Published:** 2025-10-28

**Authors:** Aoqi Dong, Xiaoying Dong, Xinying Dai, Yanru Gao, Yuewen Ning, Xiya Fan, Haiyan Liu

**Affiliations:** 1 Liaoning Key Laboratory of Chemical Additive Synthesis and Separation, Panjin Institute of Industrial Technology, Dalian University of Technology, Panjin 124221, China; aoqidong@126.com; 2 School of Petrochemical Engineering, Shenyang University of Technology, Liaoyang 111003, China; ywning11@126.com (Y.N.); 15890439402@163.com (X.F.); 3 No. 1 Oil Production Plant, Changqing Oilfield Branch of Petro China, Yan’an 716000, China; gyr2_cq@petrochina.com.cn; 4 Liaoning Key Laboratory of Chemical Additive Synthesis and Separation, Yingkou Institute of Technology, Yingkou 115014, China; lhy4486@yku.edu.cn

**Keywords:** *Suaeda salsa*, microorganisms, tea polyphenols, free amino acids, the antioxidant capacity

## Abstract

*Suaeda salsa*, an annual herb belonging to the genus *Suaeda* within the *Chenopodiaceae* family, is highly salt-tolerant and can thrive in large quantities on saline and alkaline soils. This study presents a novel fermentation technique to produce Suaeda tea, utilizing a synergistic blend of microbial agents: *Kluyveromyces marxianus*, *Komagataeibacter europaeus*, and *Acetobacter schutzenbachii*. The resulting tea demonstrates a potent antioxidant capacity, with a hydroxyl radical scavenging rate of 64.2% and an exceptional 1,1-diphenyl-2-picrylhydrazyl radical scavenging capacity of 83.3%, along with increased ferric ion reduction/antioxidant power (FRAP) reducing power (1.82), indicating its superior antioxidant profile. Through the comparison of different microbial strain combinations under varying process parameters such as fermentation temperature and duration, the experiment revealed that fermentation at 37 °C for 24 h results in the highest concentrations of tea polyphenols (TPs) (≥10.87 mg/mL) and free amino acids (26.32 mg/100 mL). The quality of the fermented Suaeda tea meets the stringent GB/T 21733-2008 standards for tea beverages, exhibiting excellent physicochemical indices and sensory attributes. The antioxidant efficacy of the fermented Suaeda tea persists significantly throughout a 180-day duration. The optimization of the fermentation process for Suaeda tea not only provides a theoretical framework for large-scale production but also establishes a foundation for *Suaeda salsa* in the tea beverage sector. This innovation enriches the market with a diverse range of health-promoting teas, catering to the growing consumer demand for nutritious and beneficial beverages.

## 1. Introduction

*Suaeda salsa* (*S. salsa*), a member of the Chenopodiaceae family and the genus *Suaeda*, has a widespread distribution along coastal regions, canal banks, and saline–alkali lowlands in provinces such as Liaoning, Heilongjiang, and Shandong in China. Suaeda possesses remarkable desalination capabilities, which aids in diminishing soil salinity and augmenting the organic matter content. The adaptability of Suaeda to saline–alkali soils underscores its ecological significance and potential for use in soil amelioration and ecological restoration initiatives [1,2,3]. Furthermore, Suaeda exhibits remarkable salt tolerance and serves as a quintessential indicator plant for saline–alkali soils. Its leaves are rich in essential amino acids and proteins, making a significant contribution to its nutritional profile [4,5,6]. It is exceptionally rich in vitamin E (up to 3310.02 mg/kg) and vitamin C (0.43 mg/g), a figure markedly surpassing that typically encountered in common leafy vegetables [7]. Its mineral profile is equally impressive, with an iron content reaching 7 mg/kg, which is significant for preventing iron-deficiency anemia, alongside substantial amounts of calcium (Ca), phosphorus (P), iron (Fe), copper (Cu), zinc (Zn), manganese (Mn), and selenium (Se) [8]. Furthermore, Suaeda contains all eight essential amino acids and a wide range of non-essential ones, and its lipid fraction consists of approximately 90% unsaturated fatty acids, of which linoleic acid accounts for 70%, making it a valuable source of omega-6 polyunsaturated fatty acids [9]. Analysis of Suaeda salsa has revealed substantial levels of flavonoids (29.78 mg/g) and polysaccharides (87.51 mg/g), underscoring its significant potential for pharmaceutical development [10].

In addition to the nutritional value, Suaeda also demonstrates medicinal properties. It contains a substantial amount of polysaccharides and flavonoid compounds, which have exhibited the capacity to bolster immune function and possess anti-aging properties [11,12]. It also shows the capacity to reduce blood sugar and lipid levels [5,13,14]. Contemporary pharmacological investigations indicate that the habitual ingestion of Suaeda has been empirically demonstrated to reduce cholesterol levels within the circulatory system and may provide a degree of prophylactic and therapeutic efficacy in addressing specific cardiovascular ailments [15,16,17]. For instance, a specific polysaccharide fraction, SuaedaP2-2, isolated from Suaeda [10,13], has demonstrated the ability to inhibit tumor cell proliferation and induce apoptosis in MCF-7 cells via the mitochondrial pathway [10]. Zheng et al. revealed that extracts from the seedlings and seeds of Suaeda significantly enhance the activity of red blood cells and inhibitory effects on both acute and chronic inflammation [18]. The polysaccharide components present in Suaeda have demonstrated considerable free radical scavenging properties, particularly against hydroxyl (•OH) and DPPH radicals. The scavenging efficacy has been observed to increase with escalating concentrations of these polysaccharides, which demonstrates its ability to slow the degeneration of bodily functions to some extent [11,19,20]. Furthermore, conjugated linoleic acid derived from Suaeda has been shown to impede weight gain and benefit gastrointestinal health, while its seed oil possesses notable lipid-reducing effects [21,22,23].

Capitalizing on Suaeda’s extraordinary biological attributes and versatility, the investigation and advancement of its applications are endowed with considerable economic worth and substantial societal importance. Tea culture boasts an extensive history within China, which has elaborate manufacturing processes [24]. Among the components present, catechins are the most plentiful and are primarily responsible for the health benefits associated with tea leaves. Research has indicated that tea polyphenols (TPs) possess a variety of beneficial properties, encompassing antioxidant, anti-atherosclerotic, anticancer, antimicrobial, antiviral, radiation protection, detoxification, weight loss, and skincare benefits [25,26,27,28]. Additionally, tea leaves are abundant in L-theanine, constituting more than fifty percent of the total amino acids found within tea [29]. The physiological effects of L-theanine are extensively documented, with studies indicating its capacity to reduce blood pressure, bolster immune response, promote a sense of calm, enhance cognitive function, and moderate the stimulating effects of caffeine [30,31,32]. To date, research on Suaeda has predominantly concentrated on plant physiology and salt tolerance, with scant attention given to its chemical composition, pharmacological activity, and the advancement of its medicinal potential. The metabolic activities of *Kluyveromyces marxianus* during fermentation lead to the production of a spectrum of volatile compounds, such as esters, alcohols, and carboxylic acids [33]. These compounds impart distinctive aroma profiles to tea, while effectively neutralizing or masking the inherent undesirable odors from the raw botanical material. *Komagataeibacter europaeus* and *Acetobacter schutzenbachii*, both characteristic acetic acid bacteria, catalyze the oxidation of ethanol to acetic acid [34,35,36]. The resulting mild acidity contributes a refreshing sensory note and acts synergistically with volatile metabolites derived from yeast fermentation to refine the overall flavor of the beverage. Furthermore, the acetic acid bacterial fermentation facilitates the generation of novel antioxidative compounds, thereby enhancing the integrated antioxidant capacity of the system [37,38].

The study conducted an experimental investigation on the fermentation of Suaeda for the purpose of tea production. Three strains of high-quality bacteria, *Kluyveromyces marxianus*, *Komagataeibacter europaeus*, and *Acetobacter schutzenbachii* were utilized. Leaves were fermented for 8, 16, or 24 h at 30 °C or 37 °C. All experiments were performed in triplicate and statistically analyzed. We quantified total tea polyphenols and free amino acids, evaluated sensory attributes, and tracked changes in physicochemical properties during the storage of the finished tea.

## 2. Materials and Methods

### 2.1. Feedstock

#### 2.1.1. The Suaeda

In this study, Suaeda was procured as the primary biomaterial, harvested from the renowned Red Beach (Panjin City, China; coordinates: 40°90421′ N, 12°82601′ E). A voucher specimen has been deposited at the Liaodong Bay New Materials Industry Science and Technology Innovation Center, Lab 503. Select leaves of Suaeda, devoid of any phytopathological traits, were meticulously cleansed with deionized water to eliminate extraneous particulate matter and surface-adherent contaminants.

#### 2.1.2. Preparation of Microbial Agent

The microbial strains used in this study, *Kluyveromyces marxianus*, *Komagataeibacter europaeus*, and *Acetobacter schutzenbachii*, were obtained from Huizao Biotechnology Co., Ltd. (Wuhan, China) [39,40].

The microbial agents, *Kluyveromyces marxianus*, *Komagataeibacter europaeus*, and *Acetobacter schutzenbachii*, were prepared separately. *Kluyveromyces marxianus* was cultured in SDB medium. The medium was sterilized at 121 °C for 15 min, and 10 mL aliquots were aseptically transferred to conical flasks under a laminar flow hood. Each flask was inoculated with 100 μL of a frozen stock culture of *Kluyveromyces marxianus* and sealed with parafilm. The cultures were then incubated in an orbital shaker at 30 °C for 48 h [41,42].

*Komagataeibacter europaeus* and *Acetobacter schutzenbachii* were cultured in a medium containing 10 g/L glucose, 10 g/L yeast extract, and 15 g/L calcium carbonate. After sterilization, 20 mL/L of anhydrous ethanol was aseptically added to the medium. Then, 10 mL aliquots of the medium were dispensed into conical flasks, and each was inoculated with 100 μL of the respective frozen stock culture. The flasks were sealed with parafilm and incubated in an orbital shaker at 30 °C for 48 h [43].

### 2.2. Fermentation Methods of Suaeda Tea

The fermentation groups were designated based on the inoculation of *Kluyveromyces marxianus* as (#1), *Komagataeibacter europaeus* as (#2), *Acetobacter schutzenbachii* as (#3), a mixture of *Kluyveromyces marxianus* and *Komagataeibacter europaeus* as (#12), a mixture of *Kluyveromyces marxianus* and *Acetobacter schutzenbachii* as (#13), a mixture of *Komagataeibacter europaeus* and *Acetobacter schutzenbachii* as (#23), and a mixture of *Kluyveromyces marxianus*, *Komagataeibacter europaeus*, and *Acetobacter schutzenbachii* as (#123) for fermentation, with the aim of investigating the optimal conditions for these seven distinct groups in the production of Suaeda tea. Non-fermented tea (CK) was prepared without the inoculation of bacterial agents. The Suaeda leaves were then inoculated with bacterial agents numbered #1, #2, #3, #12, #13, #23, and #123 at a 1% concentration for intensive fermentation to produce fermented tea.

The tea was produced following a standard fermentation process, which included wilting, killing green, inoculation, twisting, fermentation, and drying [44,45]. Preliminary experiments with fermentation times ranging from 8 h to 7 days indicated that periods exceeding 24 h led to leaf decay and deterioration. Therefore, the fermentation duration was set to 8, 16, and 24 h. The fermentation was carried out at two temperatures, 30 °C and 37 °C, to investigate the impact of temperature. All experiments were independently repeated in triplicate, and the data were subjected to statistical analysis.

### 2.3. Determination of Tea Polyphenol Content and Total Free Amino Acids in Tea

#### 2.3.1. Tea Polyphenol Content

A sample of tea weighing 0.1 g was extracted with 2.5 mL of 70% aqueous methanol (pre-warmed to 70 °C) for 10 min in a 70 °C water bath with intermittent stirring. After cooling to room temperature, the mixture was centrifuged at 3500 r/min for 10 min. The supernatant was collected, and the residue was re-extracted with another 2.5 mL of 70% methanol. The combined extracts were diluted to 5 mL with the same solvent. The solution was filtered through a 0.45 μm membrane, and 200 μL of the filtrate was diluted with 800 μL of deionized water to form the test solution. The determination of tea polyphenol content employs the ferrous tartrate colorimetric method (GB/T 21733-2008) [46].

#### 2.3.2. Total Free Amino Acids in Tea

A sample of 0.6 g of ground tea was extracted with 90 mL of boiling distilled water at 100 °C for 45 min, with intermittent stirring every 10 min. After extraction, the solution was immediately vacuum-filtered, and the residue was washed repeatedly with boiling water. The combined filtrate was transferred to a 100 mL volumetric flask and brought to volume after cooling. The total free amino acid content was determined using the ninhydrin colorimetric method (GB/T 8314-2013) [47,48].

### 2.4. Determination of DPPH Radical, •OH and Total Antioxidant Scavenging Capacity

#### 2.4.1. DPPH Radical Scavenging Capacity

Take 0.1 mL of the fermented tea to be tested in a centrifuge tube, add 3.9 mL of 0.2 mmol/L DPPH solution, shake well and leave it in a light-proof environment for 30 min, and then measure its absorbance A; take 0.1 mL of anhydrous ethanol, mix it well with 3.9 mL of 0.2 mmol/L DPPH solution and then measure its absorbance A0. Determine the absorbance under the condition of the wavelength of 517 nm.

$$K(\%)=\frac{A_{0}-A}{A}\times 100$$

where K represents the DPPH radical scavenging rate (%). The above operations were repeated three times and the average value was taken [49,50].

#### 2.4.2. • OH Scavenging Capacity

Take 1 mL of the fermented tea to be tested in a centrifuge tube, add 1 mL of 1 mmol/L FeSO_4_ solution, 1 mL of 1 mmol/L H_2_O_2_, 2 mL of 3 mmol/L salicylic acid solution, shake well, and leave it at room temperature for 30 min, determine the absorbance as AX, use deionized water to replace the salicylic acid solution to determine the absorbance A0, and use deionized water to replace the sample solution to be tested to determine its absorbance. Determine the absorbance under the condition of the wavelength of 510 nm.

$$K(\%)=1-\frac{AX\;-\;AX_{0}}{A_{0}}\times 100$$

where K represents the OH radical scavenging rate (%). The above operations were repeated three times and the average value was taken [51].

#### 2.4.3. Total Antioxidant Capacity

The principle of total antioxidant capacity determination by the FRAP method is that the antioxidant can reduce ferric-tripyridyltriazine (Fe^3+^-TPTZ) under the acidic system to produce blue Fe^2+^-TPTZ [52], and the absorbance of this substance has a maximal absorption at a wavelength of 593 nm, and the absorption peak of blue Fe^2+^-TPTZ can be determined under this condition to obtain the total antioxidant capacity of the samples to be tested. The regression equation was derived by plotting the FeSO_4_ concentration on the *Y* axis against the absorbance at 593 nm on the *X* axis, yielding Y = 1.61699X − 0.50214 with a correlation coefficient (R^2^) of 0.99591 [53]. Each sample was measured in triplicate, and results are reported as the mean.

### 2.5. Sensory Evaluation Criteria

According to the sensory evaluation protocol, infusion color, taste, and aroma were designated as the three core attributes, weighted at 30%, 40%, and 30%, respectively. A panel of twenty randomly selected assessors conducted an objective appraisal of the prepared tea samples. Each assessor scored the three attributes with reference to the descriptors and scoring criteria listed in Table 1. For each sample, the single evaluation score was calculated as the weighted sum of the three attribute scores, and the final sensory score was the arithmetic mean of the twenty independent composite scores. The rating scales and weighting scheme for the sensory attributes are detailed in Table 1 [54].

## 3. Results and Discussion

### 3.1. The pH of Fermented Suaeda Tea Inoculated with Different Microbial Agents

**Figure 1:**
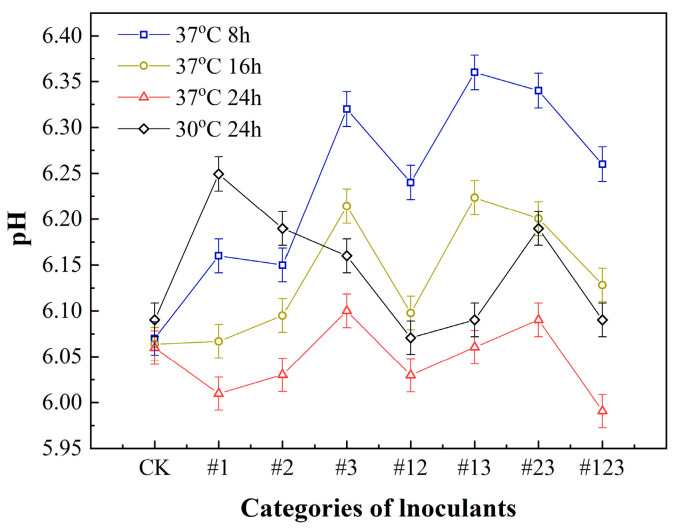
The pH value of Suaeda tea at different fermented time and temperatures.

The pH level of the tea water serves as an indicator of the tea’s taste characteristics. The pH level of the water generally falls between 5.0 and 6.7, wherein a decreased pH is associated with a more desirable taste and optimal levels of acidity and astringency for the Suaeda tea. Figure 1 shows that, during the fermentation process, the pH value of tea (CK) without inoculation was 6.06 under the condition of 37 °C and fermentation for 24 h. As fermentation increases to 24 h, the pH value of the tea gradually decreases (37 °C). The pH value of tea inoculated with #1 and #123 was the lowest, while the pH value of tea made with inoculation #3 and inoculation #23 mixed fungi was the highest. This is attributable to the metabolic processes of *Kluyveromyces marxianus* (#1), which produces organic acidic substances (acetic acid) similar to vinegar, resulting in the decrease in the pH value. Moreover, during the fermentation process, a fruit-like aroma is generated, which can effectively reduce the harshness and bitterness of the tea, greatly improving its flavor complexity and taste. *Acetobacter schutzenbachii* represents a species of diminutive, aerobic bacilli that are adept at the oxidation of alcohols and sugars, resulting in the production of acetic acid. This microorganism has the capability to employ both alcohol and sugars as carbon sources. Beyond the conversion of alcohol to acetic acid, it is also capable of oxidizing various alcohols and sugars to yield the corresponding acids and ketones. *Komagataeibacter europaeus* is predominantly isolated from fermented foodstuffs, including vinegar, black tea bacteria, and fruit juices. Contemporary research has revealed that they can facilitates the creation of non-volatile flavor compounds, such as proline, threonine, and isoleucine, within red vinegar.

### 3.2. The Content of Total Tea Polyphenols and Free Amino Acid Content Inoculated with Different Microbial Agents

**Figure 2:**
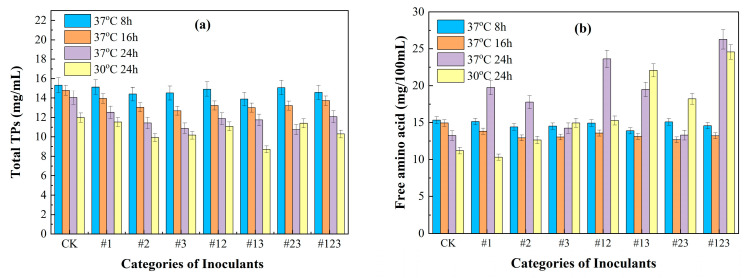
Effect of time and temperature on the content of total TPs (**a**) and free amino acid (**b**) in tea.

Figure 2a elucidates the dynamics of total TPs in the fermentation of Suaeda by different microbial treatments. A universal decrease in the theaflavin content was observed, with strains #1 and #123 displaying the least reduction, whereas strains #3 and #23 demonstrated the most pronounced depletion of total TPs. This variance corresponds to the unique polyphenolic metabolic tendencies of the microbial strains. *Kluyveromyces marxianus*, characterized by its lower total TP utilization efficiency, resulted in least total TP degradation. Conversely, *Acetobacter schutzenbachii*, with their robust decomposition capability, led to the increased consumption of total TPs. The breakdown of total TPs releases aromatic compounds, thereby enhancing the flavor profile of the tea. Nevertheless, excessive total TP reduction could undermine the nutritional integrity of the tea. Figure 2a depicts the influence of fermentation duration on the total TP content in Suaeda tea. It is observed that, as the fermentation period extends, there is a progressive diminution in the total TP content of all tea samples. It is noteworthy that there is a considerable decrease in the total TP content (24 h) compared to that of the samples fermented for only 8 h. This phenomenon is attributed to the microbial inclination to react with TPs in the absence of supplementary carbon sources, resulting in increased TP consumption during the extended fermentation period.

Figure 2b further elucidates the effect of microbial fermentation on the concentration of free amino acids on the Suaeda tea. Notably, compared to the control group’s free amino acid concentration of 13.24 mg/100 mL, the fermentation group with strains #1, #12, and #123 significantly enhanced the free amino acid concentrations to 19.74 mg/100 mL, 21.06 mg/100 mL, and 26.32 mg/100 mL, respectively. Conversely, strains #3 and #23 induced only a modest increase, reaching 14.75 mg/100 mL and 14.25 mg/100 mL, respectively. These enhancements of amino acid are ascribed to the unique metabolic attributes of the employed strains. *Kluyveromyces marxianus* exhibits exceptional proficiency in the production of free amino acids, demonstrating superior capabilities in protein hydrolysis during the fermentation process [55]. Furthermore, the synergistic interaction among the microbial species markedly enhances the levels of amino acids. Inoculation with the tri-strain blend (#123) resulted in a more pronounced enhancement of free amino acids compared to the application of individual strains, while concurrently preserving a consistent level of total polyphenol consumption. This indicates that the mixed inoculant not only promotes the production of amino acids but also ensures the preservation of other essential compounds found in tea leaves. It can be seen from Figure 2 that, under the same conditions, the total amount of free amino acids of each group fermented for 24 h was generally higher than that for 8 h. But for Groups #1, #2, and #3, which were fermented for 24 h with a single microbial community, the levels of free amino acids were slightly lower than those of the 8 h. This is due to the fact that the microbial community inoculated into the Suaeda emerged as the predominant one at this juncture. Its rapid growth led to the quick depletion of nutrients in the plant, causing a decrease in the content of free amino acids. The amino acids generated through metabolism were not enough to make up for the consumed nutrients. In the four symbiotic microbial communities (#12, #13, #23, and #123), due to the synergistic effects among different microbial species, the content of free amino acids increased significantly depend on their mutually beneficial. The production of amino acids exceeded their consumption, thereby mitigating nutrient loss and improving the tea’s flavor.

### 3.3. The Free Radical Scavenging Rate and Antioxidant Capacity of Suaeda Tea Inoculated with Different Microbial Agents

#### 3.3.1. DPPH Radical Scavenging Capacity of Suaeda Tea Inoculated with Different Microbial Agents

**Figure 3:**
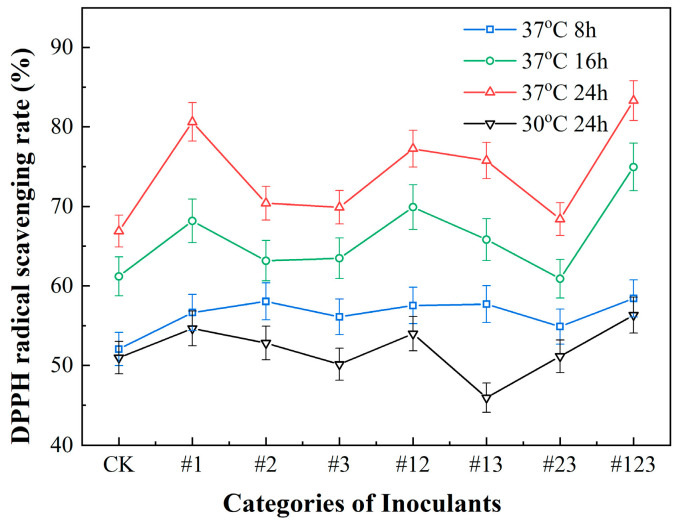
DPPH radical scavenging inoculated with different microbial agents.

Figure 3 depicts the impact of differing fermentation duration (8 h, 16 h, and 24 h) on the DPPH radical scavenging capability of Suaeda tea. The results demonstrate that the enhancement of DPPH radical scavenging capacity in Suaeda tea fermented for 8 h is not obvious, with an average scavenging rate of approximately 50%, indicating no significant distinction from the control group. Conversely, Suaeda tea (#123) fermented for 24 h exhibited a considerable enhancement of DPPH radical scavenging capacity, with rates of 83.3%. Upon conducting fermentation for a duration of 24 h, the control group (CK), which was not subjected to microbial inoculation, exhibited a mere 66.7% scavenging rate. In contrast, the tea that underwent fermentation with #1 exhibited a superior scavenging capability, reaching 80.7% at 24 h. These results imply that the DPPH radical scavenging efficacy of Suaeda tea progressively increases with prolonged fermentation duration, with the most pronounced effect noted at the 24 h interval.

Strikingly, the tea treated with the tri-strain blend (#123) further enhanced the DPPH radical scavenging rate to 83.3%. The results suggest that Suaeda tea fermented with microbial inoculants significantly enhances DPPH radical scavenging compared to the control group, indicating the positive influence of microbial fermentation on augmenting the antioxidant capacity of the tea, with the mixed-strain treatment exhibiting the most pronounced effect.

Figure 3 reveals that the results demonstrate a markedly superior DPPH radical scavenging capacity for the entire group of fermented tea incubated at 37 °C, exceeding 60% and peaking at 83.5%, and that there was an increase of 22.07% in the average rate when compared to the results achieved at 30 °C. The tea fermentation at 30 °C did not exhibit a significant disparity in DPPH radical scavenging capability compared to the control group, indicating that fermentation at this temperature exerts a limited influence on enhancing the tea’s DPPH radical scavenging capacity. Consequently, Suaeda tea fermented for a duration of 24 h at 37 °C demonstrated a considerable enhancement in DPPH radical scavenging ability. Compared to other fermented teas with antioxidant activity (such as astragalus membranaceus fermented tea), Suaeda tea exhibits a higher and more stable DPPH free radical scavenging rate [54].

#### 3.3.2. •OH Scavenging Capacity of Suaeda Tea Inoculated with Different Microbial Agents

**Figure 4:**
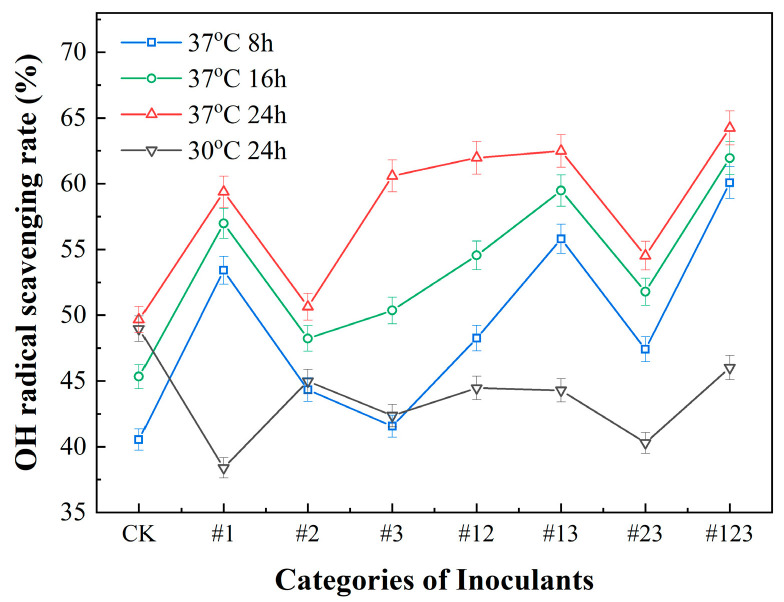
The change in •OH scavenging capacity inoculated with different microbial agents.

Figure 4 illustrates the impact of diverse microbial treatments on the hydroxyl radical (•OH) scavenging capacity of Suaeda tea. Employing uninoculated Suaeda tea as a reference control, it was discerned that the tea subjected to microbial fermentation typically exhibited a more pronounced •OH scavenging capability in contrast to the control. It is noteworthy that the control group exhibited a mere 49.6% scavenging rate at 37 °C after 24 h, whereas the tea fermented with microbial inoculant #123 exhibited the highest scavenging rate, achieving 64.2%. In contrast, the tea fermented exclusively with inoculant #2 exhibited a diminished •OH scavenging capacity, with a rate of 40.7%, which was below that of the control group at the same conditions. This suggests that fermentation employing the mixed inoculant (#123) notably augments the •OH scavenging capacity of Suaeda tea.

Figure 4 displays the impact of varying fermentation durations on the •OH scavenging capacity of Suaeda tea, with the unfermented control (CK) serving as a baseline. The experimental results distinctly show that Suaeda tea fermented for 24 h significantly outperforms the groups fermented for only 8 h in terms of the •OH scavenging capacity. Notably, the tea inoculated with #3 exhibited the most substantial difference in •OH scavenging ability, with an increase by 20% from 8 h to 24 h. Additionally, the •OH scavenging capacity of the groups inoculated with the mixed microbial inoculum (#123) fermented for 24 h demonstrated an increase by 4.1% compare to 8 h. The findings indicate that extending the fermentation time to 24 h markedly enhances the antioxidant properties of Suaeda tea, especially when employing particular strains or a combination of microbial inoculants, there is a further enhancement of the tea’s hydroxyl radical scavenging capacity.

#### 3.3.3. FRAP Scavenging Capacity Inoculated with Different Microbial Agents

**Figure 5:**
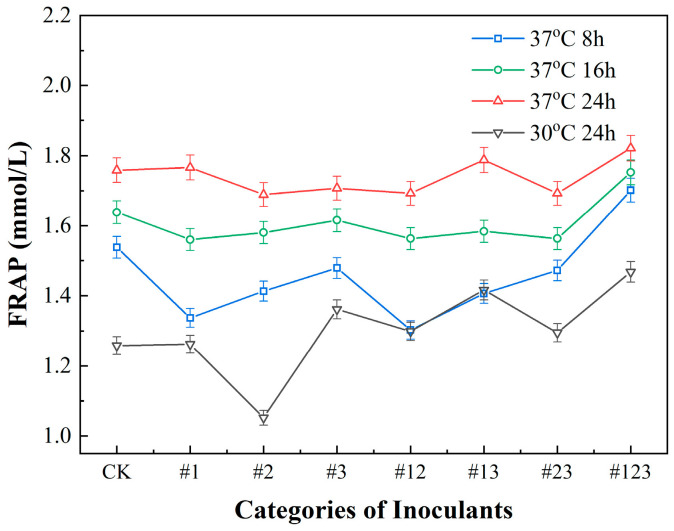
FRAP scavenging capacity of Suaeda tea inoculated with different microbial agents.

Figure 5 reveals that tea fermented at 37 °C demonstrated a superior FRAP reduction capability compared to that fermented at 30 °C. This finding emphasizes the significance of temperature as a determinant in the FRAP capacity of fermented tea, with 37 °C representing the optimal condition for achieving the greatest FRAP reduction potential.

Figure 5 presents a comparative analysis of the effect of different fermentation durations on the FRAP of Suaeda tea under same conditions. Utilizing Suaeda tea prepared without microbial inoculation (CK) as a control, the experimental findings reveal that the tea fermented for 24 h exhibits a significantly enhanced FRAP reduction capability compared to the groups fermented for only 8 h. These results underscore the importance of fermentation duration as a critical factor of the FRAP reduction capacity in fermented tea, with 24 h of fermentation emerging as the optimal condition for achieving the highest FRAP reduction potential. The results demonstrated that when the #123 strain was used for inoculation and the Suaeda tea was fermented at 37 °C for 24 h, the FRAP value of the tea reached 1.82. This value was significantly higher than that of the control group (CK, 1.76) and all the other treatments with microbial agents. Moreover, after the #123 strain was fermented at 37 °C for 8 h and 16 h, the corresponding FRAP values were 1.70 and 1.75, respectively. In contrast, when the #123 strain was fermented at 30 °C for 24 h, the FRAP value was 1.47. These outcomes imply that fermenting the Suaeda tea at 37 °C for 24 h maximizes its FRAP reduction ability, thus achieving the highest antioxidant efficacy.

### 3.4. Sensory Evaluation

**Figure 6:**
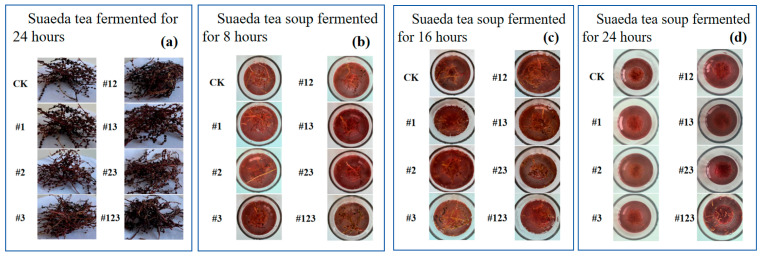
(**a**) *Suaeda liaotungensis K*, (**b**) The soup color of Suaeda tea fermented for 8 h at 37 °C. (**c**) 16 h at 37 °C. (**d**) 24 h at 37 °C.

The final fermented tea product is depicted in Figure 6a. As observed from the images, no discernible differences in the visual appearance of the tea leaves are evident after varying fermentation durations of 8, 16, and 24 h. All samples exhibit a light reddish coloration with subtle green undertones. In contrast, notable differences are observed in the clarity of the infused tea broths. Figure 6b, Figure 6c, and Figure 6d show the tea infusions brewed from leaves fermented for 8, 16, and 24 h, respectively. The broth derived from leaves fermented for 24 h (Figure 6d) demonstrates superior transparency, followed by that fermented for 16 h (Figure 6c), whereas the broth from the 8 h fermentation group (Figure 6b) appears comparatively cloudy. The smell of the fermented teas in each group is more strongly fermented and fruity than that of the non-fermented teas without the inoculation of bacterial agents, and there is no off-odor. The tea broth had a small amount of sediment after long-term storage, showed no stratification, and had a mellow taste. The specific results of the taste evaluation are presented in Table 2. Overall, after 24 h fermentation at 37 °C, the tea had a higher sensory evaluation, and its aroma was more intense. The other two groups only differed in taste in terms of individual bacteria. It can be observed that the sensory evaluation of the tea fermented by *Kluyveromyces marxianus* was significantly higher than that of other bacteria. Moreover, the tea fermented with a combination of *Kluyveromyces marxianus*, *Komagataeibacter europaeus*, and *Acetobacter schutzenbachii* possessed the most excellent taste. This indicates that *Kluyveromyces marxianus* has a positive impact on the fermentation of Suaeda tea. Upon the introduction of additional bacteria (*Komagataeibacter europaeus* and *Acetobacter schutzenbachii*), the synergistic effects among these bacteria amplify the positive influence, leading to a significant improvement in the taste of the resulting Suaeda tea.

### 3.5. Variations in the Physical and Chemical Properties of Suaeda Tea During the Storage Period

The quality of Suaeda tea was detected under the previously delineated optimal experimental conditions, Samples were provided by the mixed microbial inoculum (#123), with the fermentation process at 37 °C, 24 h. Subsequently, at an ambient temperature of 25 °C, samples were assessed at various intervals throughout the storage period (0, 30, 90, 120, 150, 180 days) to evaluate parameters including pH, total phenolic content, free amino acids, and the scavenging capabilities for DPPH radicals, hydroxyl radicals, and the FRAP. The objective was to determine the prolonged stability of the fermented tea over the storage duration, thereby offering a theoretical foundation for the processing and preservation methodologies of fermented tea.

**Figure 7:**
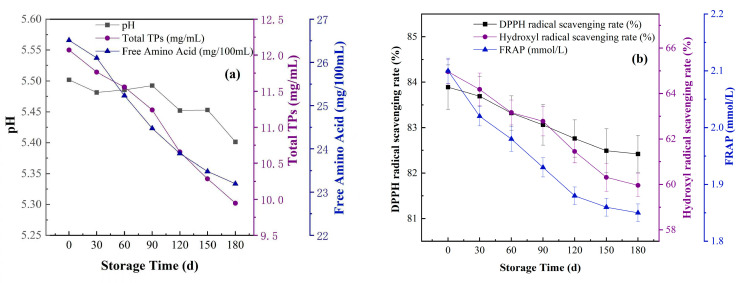
(**a**) The variation in pH value, total TP content, and free amino acid concentration of Suaeda tea during different storage periods. (**b**) The variation in DPPH radical scavenging rate, •OH scavenging rate and FRAP reduction ability of Suaeda tea at different storage periods.

Figure 7a depict the changes in physicochemical property in Suaeda tea over the storage period. With the extension of storage time, a declining trend in multiple indicators of Suaeda tea is observed. Specifically, the pH value slightly decreased from an initial 5.5 to 5.4, exhibiting minor fluctuations throughout the 180-day storage period. The content of TPs also showed a gradual decrease from 12.09 mg/mL to 9.92 mg/mL. During the first 30 to 90 days of storage, the total TPs content decreased by 0.52 mg/mL, while in the subsequent 90 to 180 days, the reduction rate increased to 1.34 mg/mL. This phenomenon suggests that the rate of decrease in total TPs content slows with prolonged storage time. Similarly, the content of free amino acids also declined, from 26.5 mg/100 mL to 23.2 mg/100 mL during 180 d, maintaining a relatively stable reduction rate. This decrease may be the oxidation of catechins [56], and the metabolic activity within the tea may also influence the levels of total TPs and free amino acids, contributing to their reduction. The alterations in the antioxidant capacity of Suaeda tea throughout the storage period are illustrated in Figure 7b. As storage duration progresses, a notable diminishment in the overall antioxidant capacity of the tea becomes apparent. The DPPH radical scavenging capacity exhibited relative stability, with a minor reduction from 83.9% to 82.5% in 180 days, suggestive of a low level of susceptibility of this metric to the length of the storage period. Conversely, the •OH scavenging capacity experienced a substantial reduction from 64.96% to 62.78% (0 d to 90 d), followed by a decrease from 62.78% to 59.96% (90 d to 180 d). Furthermore, a considerable reduction in the FRAP was noted, decreasing from 2.1 to 1.85. These empirical results suggest that although the antioxidant capacity of Suaeda tea gradually decreased as time extended, it continues to exhibit a high level of antioxidant activity even after 180 days of storage, thereby ensuring the preservation of the tea’s nutritious benefits for a prolonged period.

## 4. Conclusions

In this study, various microbial and mixed microbial agents were employed to develop a fermented Suaeda tea possessing significant nutritional value and a distinctive flavor profile. Data indicates that a fermentation duration ranging from 8 to 24 h effectively inhibited the deterioration of tea leaves and sustained the pH of the fermented tea within the optimal range of 5.98 to 6.07, thereby enhancing its palatability. Under the optimal fermentation conditions of 37 °C and 24 h, a mixed consortium (#123) composed of *Kluyveromyces marxianus*, *Komagataeibacter europaeus*, and *Acetobacter schutzenbachii* demonstrated superior fermentation results compared to other groups. In this group (#123), the hydroxyl radical scavenging rate reached 64.2%, and the DPPH radical scavenging efficiency was as high as 83.3%. Meanwhile, the resulting product had an appropriate pH value, total tea polyphenols (≥10.87 mg/mL), and free amino acid content (≥23 mg/100 mL), along with significant antioxidant properties and possessing the most favorable sensory qualities. These characteristics not only meet the demands of the health beverage market but also provide a scientific basis for the large-scale production of fermented Suaeda tea. The primary raw material offers a sustainable strategy for utilizing marginal lands and supporting agricultural diversification in coastal regions. The fermentation process established in this study provides a scalable foundation for commercial production, potentially driving rural economic development in suitable areas.

## Figures and Tables

**Table 1:** Quality factor and scoring method of tea soup.

Scoring Grade	Evaluation Criteria	Scoring Grade
Soup color (30 points)	Bright color, clear and transparent, no tea residue	25–30
	Bright color, low brightness, no tea residue	15–24
	Turbid soup, with tea residue, poor color	<14
Taste (40 points)	Mellow taste, strong tea flavor	30–40
	Moderate acidity, strong tea flavor	20–29
	Moderate acidity, weak tea flavor	<19
Aroma (30 points)	Rich and pleasant aroma	25–30
	Aroma with a hint of water vapor	15–24
	Aroma with odor	<14

**Table 2:** Assessment of Tea Quality Produced Under Various Conditions.

Sample	#1	#2	#3	#12	#13	#23	#123
37 °C 8 h	55	63	69	62	75	63	62
37 °C 16 h	67	71	62	63	78	70	65
37 °C 24 h	58	86	72	64	81	75	71

## Data Availability

The original contributions presented in this study are included in the article. Further inquiries can be directed to the corresponding author(s).
